# Ineffectual Protection in Radiology

**Published:** 1913-02

**Authors:** 


Ineffectual Protection in Radiology.—Th. Nogier of Lyon, ibid, advises the use of both leaded rubber localizers around tubes, and of protective screens, garments, etc. As examples of the danger of a false sense of security, he showed a leaded rubber skull cap, quite pervious to X rays; a glass plate sold by one of the best manufacturers are lead glass which was ordinary glass; a pair of spectacles, one lens opaque to X rays, the other transparent.

